# Clinical Outcomes of Colonic Stent in a Tertiary Care Center

**DOI:** 10.1155/2014/138724

**Published:** 2014-02-18

**Authors:** Mahesh Gajendran, Chandraprakash Umapathy, John Nasr, Andres Gelrud

**Affiliations:** ^1^Department of Internal Medicine, University of Pittsburgh, Pittsburgh, PA 15213, USA; ^2^Division of Gastroenterology, Hepatology and Nutrition, University of Pittsburgh, Pittsburgh, PA 15213, USA; ^3^The Center for Endoscopic Research and Therapeutics (CERT), Division of Gastroenterology, Hepatology and Nutrition, University of Chicago, 5700 South Maryland Avenue, MC 8043, Chicago, IL 60637-1470, USA

## Abstract

*Introduction*. Colonic obstruction is one of the manifestations of colon cancer for which self-expanding metal stents (SEMS) have been effectively used, to restore the luminal patency either for palliative care or as a bridge to resective surgery. The aim of our study is to evaluate the efficacy and safety of large diameter SEMS in patients with malignant colorectal obstruction. *Methods and Results*. A four-year retrospective review of the Medical Archival System was performed and identified 16 patients. The average age was 70.8 years, of which 56% were females. The most common cause of obstruction was colon cancer (9/16, 56%). Rectosigmoid was the main site of obstruction (9/16) and complete obstruction occurred in 31% of cases. The overall technical and clinical success rates were 100% and 87%, respectively. There were no immediate complications (<24 hours), but stent stenosis due to kinking occurred within one week of stent placement in 2 patients. Stent migration occurred in 2 patients at 34 and 91 days, respectively. There were no perforations or bleeding complications. *Conclusion*. Large diameter SEMS provide a safe method for palliation or as a bridge to therapy in patients with malignant colonic obstruction with high technical success and very low complication rates.

## 1. Background

Colorectal cancer (CRC) is the third most common cancer and second leading cause of cancer-related deaths in the Unites States. Malignant bowel obstruction (MBO) is a late complication of advanced colorectal carcinoma. Acute colorectal obstruction (ACRO) occurs in 8–29% of the colorectal cancer patients and represents a surgical emergency [1, 2]. Surgery of choice for ACRO is emergent surgical decompression with colostomy but it has been associated with significant morbidity and mortality [2–4]. Mortality rate in emergent Hartmann procedure approaches 20%, while 10–34% of the patients have stoma related complications [5, 6]. Patients presenting with bowel obstruction contribute to one-fifth of all postoperative deaths following CRC bowel surgery [7]. As a result, there has been a lot of controversy about the optimal initial therapeutic approach to patients with left-sided malignant colonic obstruction.

The first use of a self-expanding metallic stent (SEMS) for the treatment of colonic obstruction was reported by Spinelli et al. in 1992 [8]. Colonic stenting has been increasingly used as a palliative measure for patients with colorectal cancer that is not amenable to surgery and also as a bridge to curative surgery [3, 9]. In a systematic review by Khot et al., colonic stenting was found to be safe with technical and clinical success rates of 92% and 88%, respectively [3]. Experienced centers have demonstrated success rates approaching 100% in placement of colonic stents [10, 11]. Advantages of SEMS over emergent surgery include less complications, lower cost, shorter length of stay, reduced stoma rate, and decreased short-term mortality rate [12]. As a result, there has been increasing acceptance of SEMS as the first line treatment strategy [13].

There are currently four FDA-approved self-expandable metal stents (SEMS) for use in the colon [10]. Only uncovered stents have been approved because of the high rate of migration associated with partially covered SEMS when they are used in the colon [10]. The stent is removed along with the diseased bowel segment during surgery [10]. Endoscopic SEMS placement has also been used to treat colonic obstructions associated with advanced extracolonic malignancy, with comparable clinical success rates [14]. There are case reports of successful use of a retrievable covered colonic stent for treatment of a benign stricture [15].

## 2. Material and Methods

### 2.1. Patients

This study was a retrospective observational study of the patients who underwent colonic stenting for malignant colorectal obstruction in our institution from 2007 to 2010. These patients were identified by a review of the Medical Archival System (MARS). MARS is a permanent archive of both inpatient and outpatient medical records over the last 20 years. It also contains laboratory results, medication history, and radiology reports. In our study, we included all adult patients (>18 years) who underwent colonic stent placement for malignant large bowel obstruction. The data on each patient was collected from the procedure notes, radiology reports, and inpatient and outpatient clinical records.

### 2.2. SEMS Placement

**Figure 1:**
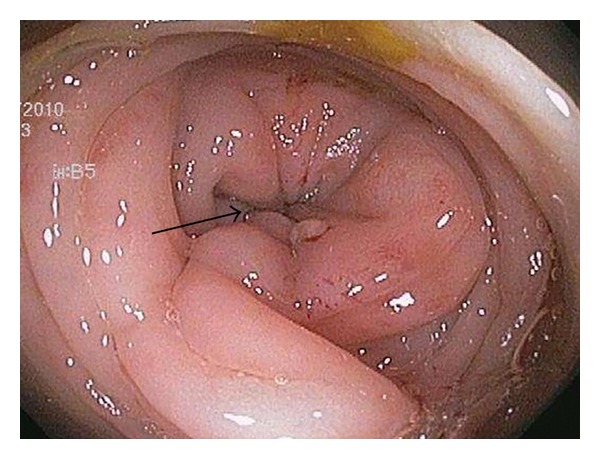
Colonoscopic view of splenic stricture (arrow) showing complete obstruction.

**Figure 2:**
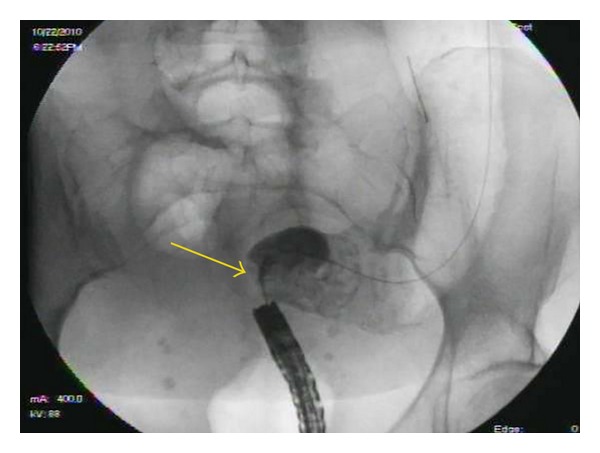
Fluoroscopic view of rectal stricture (yellow arrow).

**Figure 3:**
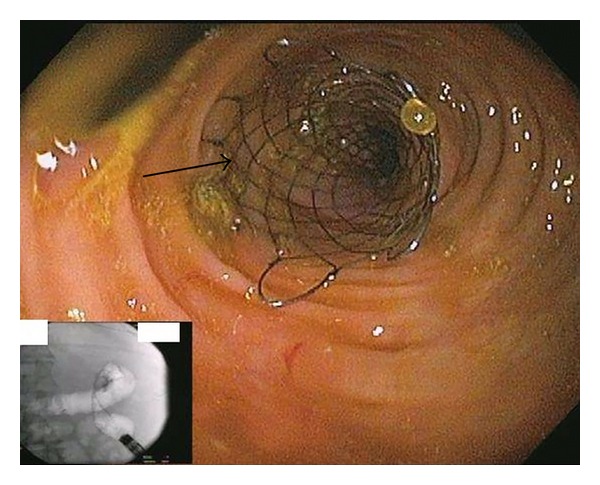
Colonoscopic view after colonic stent placement (arrow pointing at the stent, the inset shows the fluoroscopic view).

**Figure 4:**
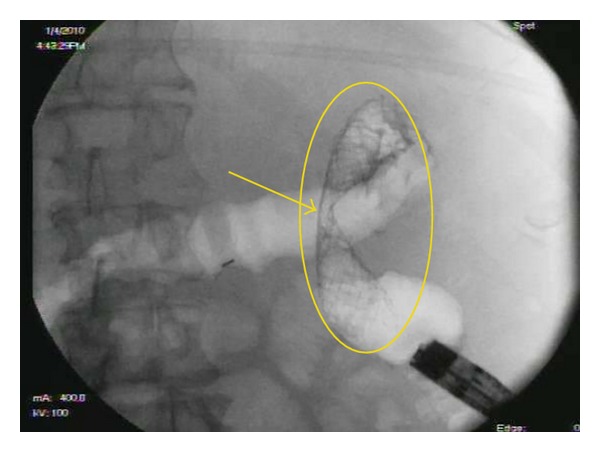
Fluoroscopic view after colonic stent placement (yellow oval).

All SEMS insertions were performed by one of the three experienced endoscopists and the technique for stent insertion was the same in all cases. Patients with distal obstruction usually underwent no cleansing preparation, while those with more proximal obstruction received a high colonic enema. The procedures were done with the patient under conscious sedation or Monitored Anesthesia Care (MAC). All stent insertions were done under fluoroscopic control. An endoscope was inserted into the rectum and advanced to the point of obstruction, which was usually identified by direct visualization of the tumor (Figure 1). Contrast was injected using an extraction balloon proximal to the tumor to determine the length of the stricture (Figure 2). The SEMS was then deployed across the stricture over the guide wire under fluoroscopic guidance (Figure 3). The endoscope was removed from the patient after confirming the placement and patency of the stent (Figure 4). Patients were then given a nonstimulant laxative (polyethylene glycol) to maintain soft stool and thus avoid impaction at the stent.

The data collected include baseline patient characteristics, indication for the SEMS placement (palliative versus bridge to surgery), outcomes, and other treatments after the SEMS placement (chemotherapy ± radiation therapy). Technical data collected include stent model, size, number of stents placed, time to perform the procedure, and the type of sedation.

### 2.3. Outcomes

Technical success was defined as accurate SEMS placement bridging the stricture with satisfactory endoscopic and fluoroscopic appearance [16]. The primary outcome of the study was clinical success, which is defined as the complete relief of the bowel obstruction, judged by clinical symptoms and/or radiographic observations within 48 hours of SEMS insertion without need for reintervention [17]. Full relief of symptoms was defined as the resolution of all subjective complaints related to the bowel obstruction as reported by the patient. Partial relief was defined as the persistence or occasional recurrence of some subjective symptoms, which could be medically managed and did not necessitate further endoscopic or surgical intervention. Clinical failure was defined as any one of the following: no relief of obstructive symptoms, objective signs of recurrence, death secondary to bowel obstruction, or need for an unplanned surgical intervention for bowel obstruction. Patency time of SEMS is the duration between the placement and the recurrence of obstructive symptoms caused by tumor overgrowth, ingrowth, or stent migration confirmed endoscopically or radiologically. When no SEMS related complications occurred, patency time was considered equal to the survival time [17]. Stent migration was defined as the feeling of stent at the anal orifice, increasing abdominal discomfort requiring further evaluation, or reobstruction with imaging revealing displacement of the stent from its previous position [16].

### 2.4. Statistical Analysis

Continuous data are described by mean, standard deviation, and range. Categorical data are presented as numbers and percentages. The time to clinical failure and death was estimated by the Kaplan-Meier product limit method. Univariate and multivariate logistic regression were used to examine the association between independent variables and 6-month survival among the patients who underwent SEMS insertion.

## 3. Results

Sixteen patients underwent SEMS placement for malignant colorectal obstruction during the study period. All the patients were referred from the Cancer Center after being evaluated by the surgical oncologist. The patients who had either complete bowel obstruction or an impending complete bowel obstruction were considered for the SEMS placement. A gastroenterologist trained in advanced endoscopy performed most of the colonic stent placements.

### 3.1. Baseline Characteristics of Patient

Table 1 outlines the baseline characteristics of the patients. The average age of the patient population was 70.8 years (range 22–101) with 56% being females. Among the patients who underwent SEMS placement, 75% of the patients were hospitalized before the procedure. The main presenting complaint was constipation (30%), bloating (25%), and vomiting (25%), with the average duration of the symptoms being 13 days. The extent and the level of bowel obstruction were confirmed using an abdominal computed tomography (69%) and colonic barium enema (12%). Interestingly 3 patients did not have any abdominal imaging within one week of the SEMS placement. At the time of stent placement, 9 of the 16 patients carried a diagnosis of colorectal carcinoma (both primary and recurrent), followed by ovarian carcinoma (3 of 16), and pancreatic carcinoma (2 of 16). Three-fourths of the patients had metastatic disease and among them 42% had peritoneal metastases, 25% liver metastases, and 8% lung metastases, while 17% had both liver and lung metastases. About 75% of the patients with peritoneal carcinomatosis had a debulking surgery done before they presented with bowel obstruction and SEMS placement. Majority of the patients (75%) were deemed to be unfit for a curative resection surgery, mainly due to the advanced stage of the cancer. The average adjusted Charlson comorbidity index score was 8. The main contributing factor for the high score was the presence of metastatic cancer and age more than 60 years.

### 3.2. Procedural Specifications

Table 2 outlines the details of procedural specifications. Seventeen stents were placed overall, with one patient requiring two stents for a long stricture. All the stents were large diameter (25-mm) stents (WallFlex stent, Boston Scientific, Natick, MA, USA). The average procedural timing was 47 minutes. For sedation, the combination of fentanyl and midazolam was used most commonly (62%), followed by Monitored Anesthesia Care (19%). The main site of obstruction was rectosigmoid area (56%) followed by the splenic flexure/descending colon (31%). On endoscopic evaluation, 31% of the obstructions were complete occlusions, while intrinsic type of compression was found in 69% of cases.

### 3.3. Technical and Clinical Success

The technical success rate was 100% and all the stent placements were performed and confirmed under fluoroscopy guidance (Table 3). The overall clinical success rate was 87.5% (14/16). Two patients had stent stenosis at days 1 and 4, respectively (Table 4). In both cases, the patients were taken for emergent endoscopy with failed attempts to dilate the stent, resulting in emergent diverting surgeries. Stent migration occurred in two patients at 34 and 91 days, respectively. Both these patients were rescoped and the migrated stent was removed. They did not require a new stent since the complete obstruction had resolved and the scope could easily be passed through the stricture area. One patient was on FOLFIRI chemotherapy and radiotherapy, while the other patient was on palliative radiotherapy. Hence the obstruction could have been resolved with the shrinkage of the tumor with a potential dilatation effect from the stent. Therefore these two patients with stent migration were not counted towards the complication rates. Out of four patients who were in the bridge-to-surgery group, three underwent curative surgical resection and one underwent emergent diverting ostomy (alive). This patient was waiting for a curative resection at the time of our data collection (3 years after stent placement). The other three patients who underwent curative resection surgery survived for 10, 21, and 28 months, respectively. The cause of death was cardiac in one patient and worsening liver metastasis in the other two patients despite chemotherapy.

### 3.4. Univariate and Multivariate Analyses Based on Survival

For the purpose of the study, we used 6-month survival from the time of stent insertion as an outcome measure. There were 7 patients who survived less than 6 months (43.75%) and 9 patients who survived more than 6 months (56.25%). On univariate analysis (Table 5), none of the measured variables predicted survival more than 6 months after the stent insertion: male sex (OR = 2.67, 95% CI: 0.35–20.51, *P* = 0.62), partial colonic obstruction (OR = 4.8, 95% CI: 0.39–58.01, *P* = 0.31), colonic obstruction beyond splenic flexure (OR = 1.33, 95% CI: 0.07–25.91, *P* = 0.85), intrinsic compression (OR = 1.25, 95% CI: 0.15–10.7, *P* = 0.84), and presence of metastasis (OR = 1.4, 95% CI: 0.144–13.6, *P* = 0.77). Also, on multivariable analysis based on binary logistic regression, none of the variables predicted survival more than 6 months after SEMS insertion in malignant colorectal obstruction: age (OR = 1.01, 95% CI: 0.93–1.07, *P* = 0.94), Charlson comorbidity index (OR = 0.91, 95% CI: 0.45–1.8, *P* = 0.78), and colonic obstruction beyond splenic flexure (OR = 0.78, 95% CI: 0.04–16.4, *P* = 0.87).

## 4. Discussion

Our study reflects the clinical outcomes of colonic stents in a tertiary care center. All the patients who had colon stent placement were reviewed without any stringent exclusion criteria. In our cohort, technical and clinical success rates of 100% and 87.5% were observed. In previous studies, colorectal stenting has been shown to have cumulative technical and clinical success rates of 94% and 91%, respectively [18–21]. The rate of success in our cohort was well within the rates of that previously reported for colorectal stenting. In our cohort, the SEMS placement eliminated the need for surgical intervention in 75% of the patients who were considered to be palliative. Palliative surgery is usually considered in patients with advanced age and presence of distant metastases and when primary carcinoma cannot be completely resected [22]. Our cohort had a mean age of 70 years with 75% having metastatic lesions, while 82% had a primary carcinoma that cannot be completely resected. This explains the high percentage of SEMS placement for palliative reasons in our cohort (75%).

**Figure 5:**
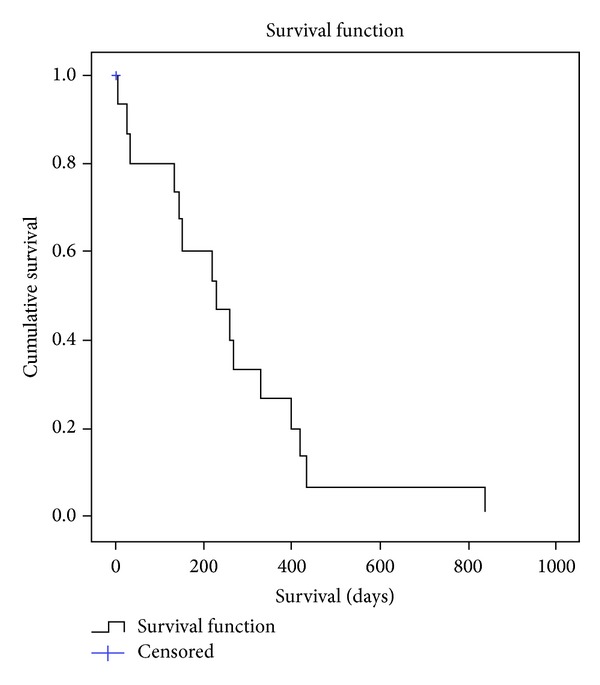
Kaplan Meier survival curve showing the survival periods of those who underwent colonic stent. The mean survival time in our cohort was 8.6 months.

Majority of the patients (15 of 16) died during the follow-up period, with a mean survival time of 8.6 months (Figure 5). Most of patients who underwent surgery in the poststent period ended up having a diverting ostomy (6 of 7; 86%). In previous studies, the operative mortality in palliative resection of colorectal cancer has been shown to be 5.6% with a crude 6-month survival rate of 71% and median survival time of 10.5 months [22]. Deans et al. reported a 5-year survival rate of less than 29% in patients who presented with colonic obstruction due to colorectal carcinoma [23]. The high mortality rate and short survival period in our study group were mainly because of the advanced stages of the cancer and most of the patients underwent the SEMS placement for palliative reasons.

Large internal diameter SEMS have been preferred to decrease the incidence of stent occlusion or migration. In our study, 12.5% of the patients (*n* = 2) had stent occlusions due to in-stent stenosis within 1 week of stent placement that resulted in need for diverting ostomy. Stent migration occurred in two patients at 1 and 3 months, respectively. The stent migration mainly resulted from shrinkage of the tumor, leading to resolution of colonic obstruction and hence did not require restenting or surgery. In a study by Luigiano et al., stent migration occurred in 2.8% of patients and occlusions in 22.8% of the cases [17]. The rectosigmoid has been reported to be the most common site for stent migration. The two most common reasons for stent occlusions include tumor overgrowth and stool impaction. The Dutch study was the only randomized trial where stage IV colorectal cancer patients were randomized to receive a WallFlex stent or to undergo palliative surgical resection. The study was stopped prematurely due to increased number of delayed perforations in the SEMS group [24]. In our study, we did not have any complications related to perforation.

The major limitation to this study is the small sample size and the retrospective nature of the study. Overall, in our experience, SEMS placement is a safe and effective treatment for management of malignant colorectal obstruction for palliative reasons.

## Figures and Tables

**Table 1:** Baseline patient characteristics (*N* = 16).

Variables	Number^a^
Age in years (±SD)	70.88 (±20)
Female gender	9 (56.25%)
Admission status	
Inpatient	12 (75%)
Same day procedure	4 (25%)
Most common obstructive symptom	
Constipation	5 (31.3%)
Bloating	4 (25%)
Overflow diarrhea	1 (6.2%)
Nausea ± vomiting	4 (25%)
Abdominal pain	2 (12.5%)
Obstruction confirmed by imaging prior to stenting	
Colonic barium enema	2 (12.5%)
CT abdomen/pelvis	11 (68.8%)
None	3 (18.7%)
Duration of symptoms (±SD)	12.8 (±9.8)
Malignancy type	
Primary colon adenocarcinoma	7 (43.8%)
Recurrent colon adenocarcinoma	2 (12.5%)
Ovarian adenocarcinoma	3 (18.7%)
Pancreatic adenocarcinoma	2 (12.5%)
Others	2 (12.5%)
Presence of known metastases	12 (75%)
Site of metastases	
Liver	3 (25%)
Lung	1 (8.3%)
Both liver and lung	2 (16.7%)
Peritoneum	5 (41.7%)
Lymph node	1 (8.3%)
Site of obstruction	
Ascending colon and hepatic flexure	1 (6.2%)
Transverse colon	1 (6.2%)
Descending and splenic flexure	5 (31.3%)
Rectosigmoid	9 (56.3%)
Type of occlusion	
Partial	11 (68.8%)
Complete	5 (31.3%)
Compression type	
Intrinsic	11 (68.7%)
Extrinsic	5 (31.3%)
Deemed fit for surgery	
Yes	3 (18.7%)
No	13 (81.3%)
Chemotherapy before stent placement	9 (56.3%)
Chemotherapy after stent placement	7 (43.7%)
Charlson comorbidity index (mean ± SD)	8.25 (±2)

^a^Values are represented as (%) for categorical variables and mean (±SD) for continuous variables.

**Table 2:** Procedural specifications.

Variables	Number (%)
Endoscopist	
A	11 (68.8%)
B	2 (12.5%)
C	3 (18.7%)
Goals of procedure	
Palliative	12 (75%)
Bridge to surgery	4 (25%)
Number of stents in one procedure	
One	15 (93.8%)
Two	1 (6.2%)
Size of stent	
25 × 120 mm	5 (31.2%)
25 × 90 mm	11 (68.7%)
25 × 60 mm	1 (6.2%)
Procedure time in minutes^a^ (±SD)	47 (±19)
Sedation	
Fentanyl + midazolam	10 (62.5%)
Meperidine + midazolam + promethazine	1 (6.2%)
Midazolam	1 (6.2%)
MAC	3 (18.7%)
Midazolam + meperidine	1 (6.2%)
ASA class	
II	8 (50%)
III	7 (43.8%)
IV	1 (6.2%)

^a^Defined by time between the first administration of sedation and the last recording of vital signs.

**Table 3:** Procedure outcomes.

Outcomes	*n* (percentage)
Technical success	16/16 (100%)
Clinical Success	
At 24 hours	15/16 (93.75%)
At 1 week	12/14 (85.7%)
At 1 month	12/14 (85.7%)
At 6 months	8/10 (80%)
till surgery/death	14/16 (87.5%)
Timing of complication	
Immediate	0
Early (<7 days)	2
Late (>7 days)	2
Type of complication	
Stent migration	2 (34 days, 91 days)
Stent stenosis	3 (1 day, 4 days)
Perforation	None
Excess bleeding	None
Use of balloon	2/16 (12.5%)
Length of stay in hospital after procedure (±SD)	3.8 days (±3.4)
Bridge-to-surgery intent outcomes (*n* = 4)	
Successful bridge to elective surgery	3
Need for emergency diverting surgery	1
Expiration prior to planned surgery	0
Subsequent interventions in palliative group (*n* = 12)	
No surgery	9
Emergent diverting surgery	2
Elective diverting surgery	1
Diverting surgery with stoma in poststent period	6 (37.5%)

**Table 4:** Patients with complications.

Patient	Complication	Days after stent insertion	Outcome
A	Stent stenosis	1	Unable to dilate the stenosis, resulting in emergent sigmoid colostomy
B	Stent stenosis	4	Unable to dilate the stenosis, resulting in emergent loop colostomy
C	Stent migration	34	Stent removed, no new stent since stricture resolved
D	Stent migration	91	Stent removed, no new stent since stricture resolved

**Table 5:** Univariate analysis to determine predictors of survival beyond 6 months after stent insertion.

Variables	Survival < 6 months	Survival > 6 months	Odds ratio	*P* value
	(*N* = 7)	(*N* = 9)		
Mean age (±SE)	71.86 (±9.4)	70.11 (±5.6)		0.87
Male sex	4 (57.1%)	3 (33.3%)	2.66	0.62
ASA class ≥ 3	3 (42.9%)	5 (55.6%)	1.67	0.61
Partial occlusion	6 (85.7%)	5 (55.6%)	4.8	0.31
Location of obstruction beyond splenic flexure	6 (85.7%)	8 (88.9%)	1.33	0.85
Intrinsic compression	5 (71.4%)	6 (66.7%)	1.25	0.84
Presence of metastasis	5 (71.4%)	7 (77.8%)	1.4	0.77
Charlson comorbidity index (±SE)	8.43 (±0.48)	8.11 (±0.84)		0.75
Chemotherapy	3 (42.7%)	4 (44.4%)	1.067	0.95
Clinical success	6 (85.7%)	8 (88.9%)	1.33	0.85
